# SHOULDER INJURY IN SURFING: A SYSTEMATIC REVIEW WITH META-ANALYSIS

**DOI:** 10.1590/1413-785220243205e279152

**Published:** 2024-10-28

**Authors:** Eduardo Bracco Cianciarulo, Tieslivi da Silva Vieira, Paulo Henrique Schmidt Lara, Paulo Santoro Belangero, Benno Ejnisman

**Affiliations:** 1. Universidade Federal de São Paulo, Escola Paulista de Medicina, Centro de Traumatologia do Esporte, Sao Paulo, SP, Brazil.

**Keywords:** Surf, Injuries, Shoulder, Water Sports, Surfe, Lesões, Ombro, Esportes aquáticos

## Abstract

Objective: To establish the epidemiological profile of shoulder injuries suffered by surfers, through the injury proportion rate, type, mechanism and/or severity, caused by surfing. Methods: This systematic review was conducted and written in accordance with the guidelines for systematic reviews– PRISMA (Preferred Reporting Items for Systematic Reviews and Meta-Analyses). The bibliographic research was carried out between January 2020 and January 2022 in journals indexed in the Web of Science, SPORTDiscus, PubMed, Scopus, Cochrane and Embase databases. Data were analyzed in RStudio, and the methodological quality of the studies was assessed. Results: Ten studies were included, all of which were retrospective in cross-sectional design and had an average methodological quality of 75%. The meta-analysis showed an injury incidence rate of 14.88%. Odds ratio analysis showed that injuries of joint origin are 7.26 times significantly higher in individuals with shoulder injuries, and injuries of bone origin and skin injuries had reduced odds of 70% and 89%, respectively. The most common mechanism of injury was the movement of paddling (57,68%), with the average prevalence of acute injuries being 31.53% and chronic injuries being 68.47%. Conclusion: There was a scarcity and/or variation in the categorization of data regarding injuries in the shoulder region resulting from surfing, with injuries of joint and musculotendinous origin being frequent; and rowing, the most overloading factor. ***Level of evidence II, Systematic Review.***

## RESUMO

Objetivo: O objetivo desta revisão sistemática foi estabelecer o perfil epidemiológico de lesões no ombro sofridas por surfistas, por meio da taxa de proporção, tipo, mecanismo e/ou gravidade de lesões ocasionadas pela prática do surfe. Métodos: A presente revisão sistemática foi conduzida e redigida de acordo com as diretrizes para revisões sistemáticas – Prisma (Preferred Reporting Items for Systematic Reviews and Meta-Analyses).A pesquisa bibliográfica foi realizada entre janeiro de 2020 e janeiro de 2022, nos periódicos indexados nas bases de dados Web of Science, SPORTDiscus, PubMed, Scopus, Cochrane e Embase. Os dados foram analisados no RStudio, além disso também foi avaliada a qualidade metodológica dos estudos. Resultados: 10 estudos foram incluídos, sendo todos de delineamento transversal retrospectivos e de qualidade metodológica média de 75%. A meta-análise apresentou uma proporção de incidência de lesões de 14,88%. A análise de razão de chances mostrou que lesões de origem articulares são 7,26 vezes significativamente maiores em indivíduos com lesões no ombro, e lesões de origem óssea e as lesões na pele apresentaram chances reduzidas em 70% e 89%, respectivamente. O mecanismo de lesão mais comumente relatado foi o movimento da remada (57,68%), sendo a prevalência média de lesões aguda de 31,53%, e de lesões crônicas 68,47%. Conclusão: Observou-se uma escassez e/ou variação de categorização de dados referentes às lesões na região do ombro decorrentes do surfe, sendo frequentes lesões de origem articular e musculotendínea; a remada foi considerada o principal fator de sobrecarga. ***Nível de Evidência II, Revisão Sistemática.***

## INTRODUCTION

 Surfing is a water sport that has become increasingly popular in recent years. IBRASURF estimates that there are three million surfers in Brazil and over 35 million worldwide, with an annual growth rate of 11.5%. ^1^ ^,^ ^2^ Global participation is expected to increase even further following the sport’s debut at the Tokyo Olympics in 2021, as well as medical and scientific interest in the sport. ^3^ The increasing global spread of surfing is accompanied by an increase in the level of competition and consequently the frequency of injuries, which reinforces the need to understand the pathogenesis. 

 Studies conducted with competitive and recreational surfers have shown an overall incidence rate of 0.74 to 1.79 injuries per 1,000 hours of surfing. ^4–6^ In a review looking at acute injuries in surfers, it was noted that injuries to the head, face and neck were the most common and that impact with the surfboard was the most common mechanism. ^6^ More recently, a new study by the same group, focusing on gradually developing conditions that became chronic, found that the most commonly reported injury sites were the spine at 29.3%, the shoulder at 22.9% and the head, face and neck at 17.5%, with the most common mechanism being paddling, which accounted for 37.1% of injuries. ^3^

 Time and motion analyses have shown that surfing is an intermittent sport and that part of the time, on average 51% (25–70%), is spent paddling. ^7^ Therefore, it is reasonable to assume that overuse injuries in the shoulder are related to the repetitive motion of the paddle stroke and the body position while paddling. ^8^ In addition, the paddling movement can lead to muscular imbalances and thus impair joint movements. ^9^ ^,^ ^10^

 Although the shoulder is related to the most time-consuming activity in surfing and is the region where most surgical procedures are performed, there is little research that examines and summarizes these data. ^2^ ^,^ ^11^

Given the significant growth of this sport and the lack of specific studies on its injuries, there is a recognized need for research such as this study to estimate the proportion of injuries, types, mechanisms and/or severity of shoulder injuries.

## MATERIALS AND METHODS

 This study was written according to the PRISMA (Preferred Reporting Items for Systematic Reviews and Meta-Analyzes) guidelines. ^12^ The protocol was published in the PROSPERO registry (CRD42021252228). 

### Search strategy

 The search was conducted between January 2020 and May 2022 in journals indexed in the Web of Science, SPORTDiscus, PubMed, Scopus, Cochrane and Embase databases. The different search syntaxes are listed in Appendix 1 and can be accessed at https://www.crd.york.ac.uk/PROSPEROFILES/252228_STRATEGY_20210429.pdf . 

All records were imported into the Mendeley management software and duplicate publications were removed. The articles in question were also identified by bibliographic linking.

### Inclusion and exclusion criteria

The inclusion criteria were: (1) studies involving surfers of any age, both genders, and any experience level; (2) studies that mentioned surfing-related injuries; (3) studies that categorized shoulder-specific injuries; (4) studies that reported at least one of the following outcomes: Frequency of shoulder injury, types of injuries, severity, and/or mechanisms; (5) Studies published between 2000 and 2021. There was no restriction in the search: injury stage, language or study design.

Exclusion criteria were studies that: (1) focused on different water sports such as wakeboarding, water polo, or water skiing; (2) did not include surfing injuries; (3) categorized the shoulder along with other upper limb regions such as the elbow or arm or used only general terms such as upper limb; (4) were reviews or secondary analyses; (5) were incomplete or did not include sufficient data on the outcomes of interest.

### Data extraction

Two independent reviewers used Rayyan software to check the results for selecting eligible studies against the pre-specified inclusion and exclusion criteria. Discrepancies were discussed with a third reviewer.

### Assessment of methodological quality and risk of bias

 The risk of methodological bias was assessed using the Appraisal for Cross-Sectional Studies (AXIS tool). ^13^ The choice of tool followed the current recommendations on evidence-based medicine and methodological quality. ^14^

### Data analysis

For the type of injury, a meta-analysis was performed to estimate the odds ratio (OR) of the dichotomous outcome “shoulder injuries” versus “injuries in other regions” by injury subgroup. Due to the variety of injury type names and presentations, common occurrences were grouped into: (1) “articular”: ligament strains, cartilage damage, dislocations, subluxations; (2) “musculotendinous”: strains, sprains, inflammation, and ruptures; (3) “bone”: fractures and others (avulsions, bone edema); (4) “skin injuries”: Lacerations, abrasions, contusions and wounds; (5) “Nerve injury”: Nerve compression, stretching or other; (6) “Other”: not identified by the study.

For severity and mechanism, the relative frequencies were summed and divided by the absolute sample to obtain the total frequency. Data were analyzed with R software (v.4.0.5) and RStudio (v.1.4.1106) using the ‘metaprop’ and ‘metabin’ packages.

## RESULTS

### Study selection

**Figure 1.**
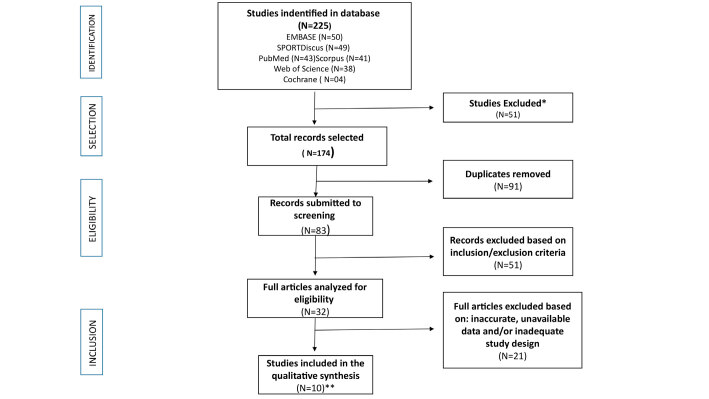
Flowchart of the article search in databases. (*Not within the specified search limits; **Two included studies were combined as they included the same cohort of individuals).

 A total of 225 studies were identified using the search terms. After applying the inclusion and exclusion criteria, the included studies are shown in Figure 1 . 

### Analysis of included studies

 The included studies were retrospective cross-sectional studies published between 2004 and 2021. Four studies were from Australia, two from the United States and one each from the United Kingdom, Japan, New Zealand and Brazil. After quality assessment, six were rated as ‘good’ and four as ‘fair’,” with an average score of 75% (SD ±7.82%), ranging from 65% to 85%. The final results are in Table 1 . 

 In terms of data collection, only one study strictly referred to shoulder injuries, while the other nine studies quantified the total number of injuries and categorized the location according to the region of occurrence, with shoulder being one of them. ^15^ Four studies used medical records, ^11^ ^,^ ^16^ ^-^ ^18^ while the other five used questionnaires administered by the research group either online or by interview. ^2^ ^,^ ^4^ ^,^ ^18^ ^-^ ^21^ Only one study collected data in person through standardized physical assessments after the online application. ^15^

 Overall, 70% of the studies provided information on the type of injury, ^4^ ^,^ ^11^ ^,^ ^15^ ^,^ ^16^ ^,^ ^19^ ^,^ ^20^ and only 20% reported on the mechanism of injury occurrence. ^2^ ^,^ ^4^ In terms of injury severity, 80% of studies followed some criteria for grouping events, although inclusion criteria and definitions varied. 

 The average age of the surfers was 34.6 years with a standard deviation of 12.4 years. The gender distribution was 85.6% male and 14% female. Only three of the included studies did not specify the level of experience of the surfers, two considered a large proportion of the cohort to be recreational surfers, three restricted the study to professionals, one to amateurs, while the last specified as a criterion people who had at least 12 months experience. The descriptive characteristics are listed in Table 1 . 

Based on the evaluation of the included studies, a total of 5,201 surfing practitioners were observed, with 3,280, approximately 63%, experiencing some type of injury. Of these, 519 were shoulder injuries.

**Figure 2.**
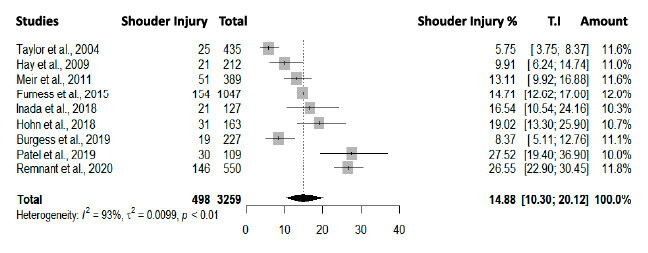
Forest plot comparison of shoulder injuries in surfing practitioners. The columns present the studies sequenced by year; number of shoulder injuries; total injuries in studies; shoulder injuries %; 95% CI; study weight in the overall meta-analysis.

 For the analysis of relative and absolute proportions, a study with *outlier* results, which evaluated a population of surfers exclusively with shoulder injuries, was excluded. ^15^ Thus, a meta-analysis with nine included studies (n = 498), weighting injury frequencies by study size, is presented in Figure 2 . 

 The summary of the meta-analysis showed an incidence of shoulder injuries of 14.88% (95% CI, 10.30 - 20.12). The lowest proportion of shoulder injuries in relation to total injuries was observed in the oldest study by Taylor et al., ^19^ with 5.75% (95% CI, 3.75 – 8.37). The highest proportions were found in more recent studies, with 26.55% (95% CI, 22.90 – 30.45) in Remnant et al. ^2^ , and 27.52% (95% CI, 19.40 – 36.90) in Patel et al. ^18^ The heterogeneity between them, as measured by the I ^2^ statistic, was significant at 93%. 

### Types of injuries

 Broad classifications were used to simplify the amount of data on the type of injuries. Of the ten studies, only seven reported the type of occurrence in their sample, representing an absolute total of 326 shoulder injuries. ^4^ ^,^ ^11^ ^,^ ^15^ ^,^ ^16^ ^,^ ^18^ ^-^ ^20^ Joint injuries accounted for 51% (n = 166), muscle-tendon injuries 30% (n = 97), bone injuries 4% (n = 14), skin injuries 3% (n = 11), nerve injuries 3% (n = 10) and unspecified, ‘other’ 9% (n = 28). 

**Figure 3.**
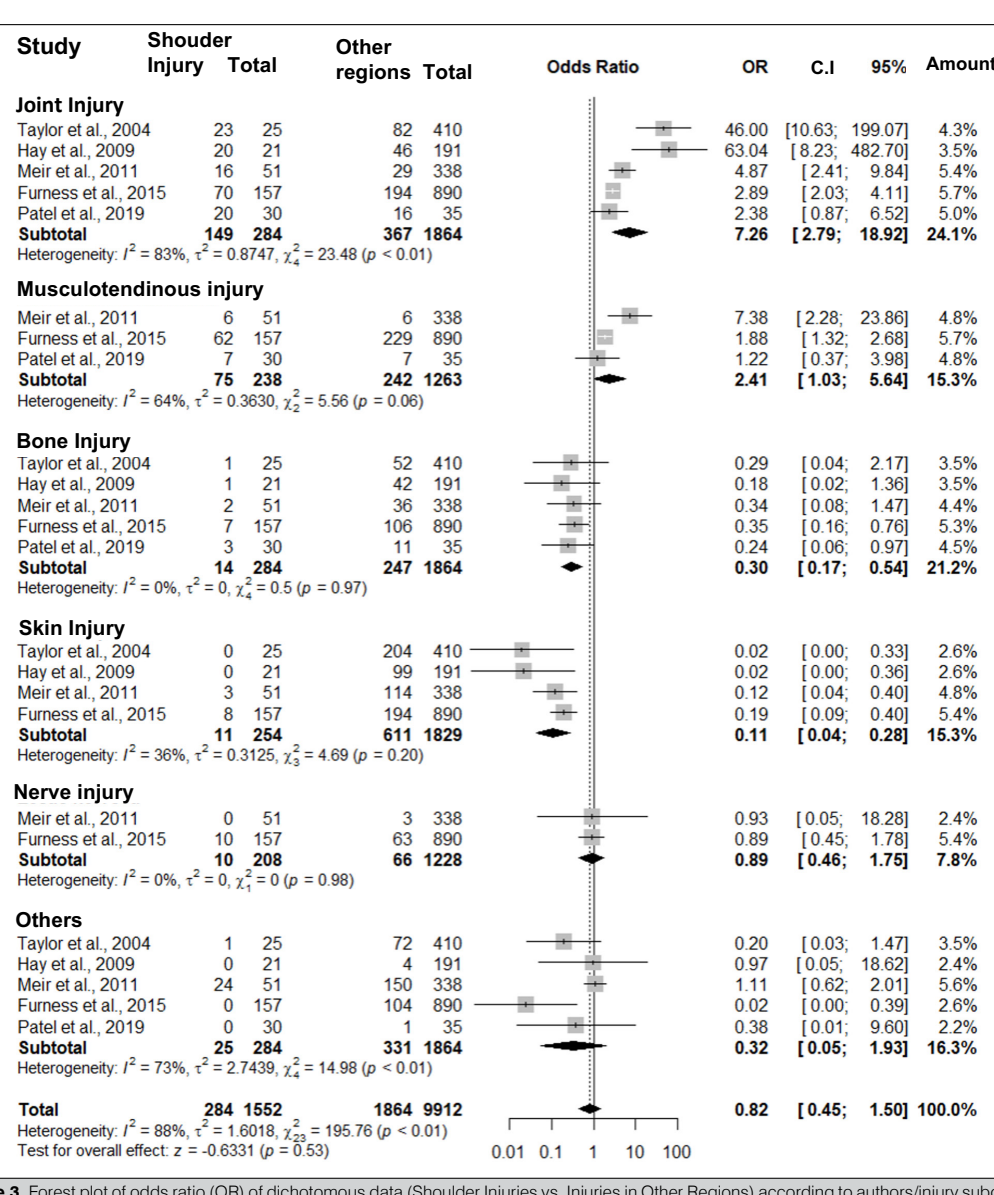
Forest plot of odds ratio (OR) of dichotomous data (Shoulder Injuries vs. Injuries in Other Regions) according to authors/injury subgroup.

 Dichotomous random-effects analyses were performed to examine whether the odds ratios (ORs) of individuals with shoulder injuries and those with injuries in other regions, i.e. without the outcome of interest, had similar distributions with respect to the type of injury event. The analysis is shown in Figure 3 . 

The meta-analysis showed that individuals with shoulder injuries were 7.26 times more likely to have joint-related injuries than those with injuries in other regions (OR 7.26; 95% CI 2.79 – 18.92; p = 0.0001). The likelihood of muscle-tendon injuries was also 2.4 times higher in people with shoulder injuries, without statistical significance (OR 2.41; 95% CI 1.03 – 5.64; p = 0.06). In contrast, the likelihood of bone and skin injuries was reduced by 70% and 89%, respectively, in people with shoulder injuries (OR 0.30; 95% CI 0.17 – 0.54; p < 0.0001 / OR 0.11; 95% CI 0.04 – 0.28; p < 0.00001).Nerve injury and other species were on the zero line, hence a similar relationship (OR 0.89; 95% CI 0.46 – 1.75; p = 0.75 / OR 0.32; 95% CI 0.05 – 1.93; p = 0.22). The summary showed that the probability of occurrence in the shoulder region or in other regions was the same (OR 0.82; 95% CI 0.45 – 1.50; p = 0.53). However, heterogeneity was significant (p < 0.00001), a factor indicating low quality of the cohort.

**Table 1.** Assessment of methodological quality and descriptive characteristics of the included studies

Author, year		AXIS tool points	Quality assessment	Data collection method	Population demographics			Number of participants (N)			Total shoulder injuries/total study injuries	Competition level	
				Average Age (X ± SD)		Sex (M/F%)							
Taylor et al., 2004 ^19^		15/20	Good	Interview Questionnaire	28.2 ± 7.9		90.2/9.8	Research: 646			13/168	N/D	
						Emergency: 267			12/267				
Hay et al., 2009 ^16^		13/20	Fair	Medical Records	27		80/20	212			21/212	N/D	
Meir et al., 2011 ^20^		17/20	Good	Online Survey	31.7 ± 12.85		85.4/14*	685			51/389	71.5% recreational	
Furness et al., 2015 ^4^		16/20	Good	Online Survey	35.8 ± 13.1		91.3/8.7	1348			154/1047	Active surfers with at least 12 months experience	
Inada et al., 2018 ^20^		13/20	Fair	Medical Records	N/D		N/D	Championships and Clinic: 65			4/65	Professional	
						Clinic: 62			17/62				
Hohn et al, 2018 ^11^		17/20	Good	Medical Records	28.5		92.6/7.4	86			31/163	Professional	
Burgess et al, 2019 ^21^		15/20	Good	Online Survey	35 ± 13.2		77/23	227			19/227	Professional	
Patel et al, 2019 ^18^		14/20	Fair	Medical Records	36		74/26	109			30/109	N/D	
Remnant et al, 2020 ^2^		16/20	Good	Online Survey	34.6 ± 11.9		82/18	1473			146/550	63% recreational	
Gomes et al., 2020 ^15^		13/20	Fair	Online Survey	28 ± 5		100/0	21			21/21	Amateur athletes	
**Average**	**75 % (SD ±7.82%)**				**34.6 ± 12.4**	**85.6/14**			**5201**	**519/3280**			

N/D: Not declared; *Meir et al., 2011 reported a total of 0.6% (4) transgender individuals participating in the survey.

### Mechanism of injury

 Two studies directly reported the mechanism of injury by region, totaling 345. ^2^ ^,^ ^4^ Rowing motion was the most commonly reported, followed by injuries related to maneuvers and contact or direct trauma injuries. The others include duck jumping and unknown causes. 

### Severity

 Eight articles reported on the severity of injuries, with widely varying classification criteria. Three studies divided them into chronic and acute. ^2^ ^,^ ^17^ ^,^ ^19^ Two others differentiated them according to surgical or non-surgical intervention. ^11^ ^,^ ^18^ Hay et al. ^16^ and Furness et al. ^4^ analyzed the incidences as mild or severe, with different criteria. Gomes et al. ^15^ quantified pain intensity using a numerical scale (NRS). Due to the notable differences in definitions/nomenclature and details, it was decided to stratify the cases as reported and group only the similar cases. The severity of the injuries is shown in Table 2 . 

Four methods of distinguishing the severity of injury were observed in the studies. Due to the different classifications, only the subgroup that distinguished between acute and chronic injuries was suitable for comparison. We observed 31.53% acute injuries and 68.47% chronic injuries.

**Table 2.** Frequency of injury severity according to categorization and definitions.

**Author, Year**	**Shoulder Injuries % (N)**		**Definition Described by the Study**
	**Acute**	**Chronic**	
Taylor et al., 2004 ^22^	40% (10)	60% (15)	Acute: Individual treated by someone other than the surfer or time off activities.
			Chronic: Disorders not related to an acute injury requiring treatment.
Inada et al.,2018 ^20^	19% (4)	81% (17)	Acute: Injuries treated in outpatient care.
			Chronic: Non-surgical treatments such as medication or physical therapy.
Remnant et al.,2020 ^2^	35.6% (52)	64.4% (94)	Acute: Injury duration less than 3 months.
			Chronic: Injury duration of 3 months or more.
	**Surgical**	**Non-Surgical**	
Hohn et al.,2020 ^11^	73% (20)	27% (7)	Surgical: An acute injury due to a traumatic event with primary surgical treatment.
			Non-surgical: An acute injury due to a traumatic event or a chronic injury due to overuse with non-surgical treatment.
Patel et al.,2019 ^25^	23.3% (7)	76.7% (23)	Surgical: Surgical procedure data that has been entered into the electronic medical record system.
			Non-surgical: Injuries caused by trauma in patients who presented to the facility less than 6 months after the surfing injury.
	**Severe/**	**Mild**	
Hay et al., 2009 ^19^	4.5% (1)	95.5% (21)	Mild: patients who could be discharged after treatment, i.e. injuries that did not require hospitalization.
			Severe: Injuries requiring hospitalization.
Furness et al., 2015 ^4^	78.6% (121)	21.4% (33)	Mild: Did not interfere with work or surfing or did not require treatment by medical professionals.
			Severe: The participant required one or more days off work and/or surfing and/or required treatment by a medical professional.
	Pain Yes (NPS)*	Pain No (NPS)*	
Gomes et al.,2020 ^18^	42.9% (9)	57.1% (12)	The intensity of shoulder pain at the time of data collection was assessed using the Numerical Pain Scale (NPS).

* NPS: Numerical Pain Scale. ** SD: Scapular dyskinesia.

## DISCUSSION

 he proportion of shoulder injuries compared with other regions was 14.88% (95% CI, 10.30-20.12), depending on the size of the studies, and ranged from 5.75% (95% CI, 3.75–8.37) in Taylor et al, 2004 ^19^ , to 27.52% (95% CI, 19.40 – 36.90) in Patel et al ^18^ . The variability in incidence can be attributed to different data collection methods or the level of experience of the surfers. 

 The percentage increase in injuries probably reflects the increasing global spread of surfing and the simultaneous increase in the level of competition. The complexity of the maneuvers and the difficulty of execution have increased. ^22^ One of the most famous maneuvers is the aerial, which is associated with high performance and high risk. ^23^ In addition, advances in design and materials have led to lighter, smaller boards that improve performance. 

 Furness et al. ^4^ studied 194 surfers who regularly perform aerial maneuvers, from which 94 suffered serious acute injuries over a 12-month period. The author also found a significant increase in serious injuries in this group, with an increased strain on ligaments and contractile tissue and an increase in muscle and joint injuries. According to Bickley et al, ^24^ riskier maneuvers or larger waves contribute to higher injury rates in professionals. 

 The high incidence of shoulder joint injuries, which is 7.26 times higher than in other regions, and of muscle-tendon origin injuries, which is 2.4 times higher than in other regions, is attributed to the overload caused by the repetitive strokes and the body posture during paddling. The causes are thought to include hyperextension of the cervical and lumbar spine, continuous isometric contraction of the neck and scapular muscles, and medial rotation during paddling. ^7^

 Furness et al, ^25^ who examined the strength profile of medial and lateral rotation, concluded that professionals have greater strength in the medial rotation muscles than in the lateral ones. When identifying an asymmetry between the sides for the lateral rotators, the non-dominant arm was weaker. It is therefore hypothesized that paddling promotes unbalanced muscle development that may lead to scapular dyskinesia (SD). ^9^ Gomes et al. ^15^ who studied the prevalence of SD concluded that it was present in 71.4%, with a higher prevalence of dyskinesia with a protrusion at the medial edge of the scapula (57.1%), and that 23.8% had SD at rest. Decreased thoracic extension alters the risk of dyskinesia of the scapula and increases the risk of impingement around/d the glenohumeral joint. ^8^

 There is also an increase in overuse injuries as surfers are surfing more frequently and for longer periods in wetsuits that insulate the body in cold water. ^26^

 A systematic review of the epidemiology of injuries found that skin injuries (abrasions, lacerations, burns, hematomas, bruises) were the most common at 46% ^6^ . In the same study, the most frequently injured region was the head (33.8%), followed by the lower limbs (33.0%). Arm injuries accounted for 16.5%. ^6^

 The most reported mechanism was paddling (57.68%), followed by maneuvers (14.49%). It is estimated that 54% of surfing time is spent paddling, which would explain the propensity for shoulder injuries. ^27^ Another factor that may be associated with paddling injuries is the type of board. Remnant et al. ^2^ concluded that longboards pose a greater risk than shortboards, possibly because longboards require a greater elbow angle and greater abduction of the shoulder during recovery to avoid collision with the edge. ^2^

 Studies on the mechanism of injury found that the most common cause was collision between surfer and board, followed by approaching the wave or performing a maneuver. ^6^ ^,^ ^28^ Subsequently, when Hanchard et al. ^3^ studied the same population but for chronic injuries, they found a higher percentage caused by paddling. This result suggests a link between paddling-related injuries and chronic disease. 

 Nathanson et al. ^29^ reported that musculoskeletal injuries account for 60% of chronic surfing injuries, with the shoulder being the most affected joint. It is concluded that chronic injuries of gradual onset related to the rotator cuff are more common in surfers due to the repetitive nature of paddling. ^30^ This analysis is supported by Remnant et al, ^2^ who found rotator cuff injuries and/or bursitis in 80% of individuals. 

 In terms of injury severity, only acute and chronic injuries were comparable, with chronic injuries being twice as common as acute injuries. A further classification of severity would be surgical or non-surgical. Hohn et al. ^11^ described that shoulder injuries were the most operated on (73%) compared to other regions. These were usually instability, rotator cuff or SLAP lesions. Similarly, Patel et al. ^18^ observed surgical intervention in cases of anterior shoulder dislocation with persistent symptoms of instability, rotator cuff, SLAP lesion, traumatic osteolysis of the acromioclavicular joint and chondral shear injury of the humeral head. 

Compared to other modalities, surf training is not yet sufficiently developed and widespread.

 This review was limited by the availability of studies with only retrospective cross-sectional designs. Another limiting factor lies in the method of data collection: an online questionnaire that depends solely on the participants’ memory and is therefore subject to recall errors. The recall rate decreases with increasing detail, with accuracy decreasing by up to 61%. ^31^ ^,^ ^32^ In addition, injured surfers are more likely to respond to surveys than uninjured surfers, leading to inaccurate incidence rates. ^6^

Although hospital records were collected in four studies, there was no consistency in reporting professional surfing experience, a factor that could improve diagnostic accuracy and injury presentation. In addition, the cohort of patients included in this study provides greater statistical power, which facilitates validation of conclusions.

## CONCLUSION

There is little data in the literature on shoulder injuries during surfing. The incidence of shoulder injuries compared to other regions is 14.88%, with joint and muscle-tendon origins being more common. These incidences can be attributed to overuse of the shoulder due to the repetitive paddling action and posture of paddling, suggesting that the practice of this sport appears to promote imbalanced muscle development.

Further research focusing exclusively on sports practitioners is needed.

## **Appendix 1.** search syntax used in databases.

**Table:**

Databases	Search strategy	Total
**Embase**	(‘injuries’/exp OR ‘injuries’ OR ‘athletic injuries’/exp OR ‘athletic injuries’ OR ‘lesion’/exp OR ‘lesion’ OR ‘biomechanical phenomena’/exp OR ‘biomechanical phenomena’ OR ‘cumulative trauma disorders’/exp OR ‘cumulative trauma disorders’ OR ‘ligaments, articular/injuries’ OR ‘lacerations’/exp OR ‘lacerations’) AND(‘shoulder’ OR ‘shoulder injuries’ OR ‘shoulder pain/physiopathology’ OR ‘shoulder pain/epidemiology’ OR ‘shoulder pain/etiology’ OR ‘rotator cuff injuries’ OR ‘shoulder dislocation’ OR ‘shoulder fractures’ OR ‘bankart lesions’ OR ‘shoulder impingement syndrome’) AND(‘surfing water sport’/exp OR ‘surfing water sport’ OR ‘surf’ OR ‘surfing’/exp OR ‘surfing’ OR ‘recreational water’/exp OR ‘recreational water’ OR ‘recreational athlete’/exp OR ‘recreational athlete’ OR ‘surfing (water sport)’/exp OR ‘surfing (water sport)’)	**50**
**SPORTDiscus**	All_fields:((“Injuries” OR “Athletic Injuries” OR “Lesion” OR “Biomechanical Phenomena” OR “Cumulative Trauma Disorders” OR “Ligaments, Articular/injuries” OR “Lacerations”)) AND all_fields:(“Shoulder” OR “Shoulder Injuries” OR “Shoulder Pain/physiopathology” OR “Shoulder Pain/epidemiology” OR “Shoulder Pain/etiology” OR “Rotator Cuff Injuries” OR “Shoulder Dislocation” OR “Shoulder Fractures” OR “Bankart Lesions” OR “Shoulder Impingement Syndrome”)) AND title:((“Water Sports” OR “Wave Surfing” OR “Surfing, Wave” OR “Surfboarding” OR “Surfing” OR “Surf”)	**49**
**Pubmed**	(“Injuries”[All Fields] OR “Athletic Injuries”[All Fields] OR “Lesion”[All Fields] OR “Biomechanical Phenomena”[All Fields] OR “Cumulative Trauma Disorders”[All Fields] OR “ligaments articular injuries”[All Fields] OR “Lacerations”[All Fields]) AND (“Shoulder”[All Fields] OR “Shoulder Injuries”[All Fields] OR “Shoulder Pain/physiopathology”[All Fields] OR “Shoulder Pain/epidemiology”[All Fields] OR “Shoulder Pain/etiology”[All Fields] OR “Rotator Cuff Injuries”[All Fields] OR “Shoulder Dislocation”[All Fields] OR “Shoulder Fractures”[All Fields] OR “Bankart Lesions”[All Fields] OR “Shoulder Impingement Syndrome”[All Fields]) AND (“Water Sports”[All Fields] OR “Wave Surfing”[All Fields] OR “Surfboarding”[All Fields] OR “Surfing”[All Fields] OR “Surf”[All Fields])	**43**
**Scopus**	( ALL ( ( “Injuries” OR “Athletic Injuries” OR “Lesion” OR “Biomechanical Phenomena” OR “Cumulative Trauma Disorders” OR “Ligaments, Articular/injuries” OR “Lacerations” ) ) ) AND ( ALL ( ( “Shoulder” OR “Shoulder Injuries” OR “Shoulder Pain/physiopathology” OR “Shoulder Pain/epidemiology” OR “Shoulder Pain/etiology” OR “Rotator Cuff Injuries” OR “Shoulder Dislocation” OR “Shoulder Fractures” OR “Bankart Lesions” OR “Shoulder Impingement Syndrome” ) ) ) AND ( TITLE-ABS-KEY ( ( “Surfboarding” OR “Surfing” OR “Surf” ) ) )	**41**
**Web of Science**	“(”“Injuries”” OR ““Athletic Injuries”” OR ““Lesion”” OR ““Biomechanical Phenomena”” OR ““Cumulative Trauma Disorders”” OR ““Ligaments, Articular/injuries”” OR ““Lacerations”“) AND (”“Shoulder”” OR ““Shoulder Injuries”” OR ““Shoulder Pain/physiopathology”” OR ““Shoulder Pain/epidemiology”” OR ““Shoulder Pain/etiology”” OR ““Rotator Cuff Injuries”” OR ““Shoulder Dislocation”” OR ““Shoulder Fractures”” OR ““Bankart Lesions”” OR ““Shoulder Impingement Syndrome”“) AND (”“Water Sports”” OR ““Wave Surfing”” OR ““Surfing, Wave”” OR ““Surfboarding”” OR ““Recreation”” OR ““Surfing”” OR ““Surf”“)”	**38**
**Cochrane**	((“Injuries” OR “Athletic Injuries” OR “Lesion” OR “Biomechanical Phenomena” OR “Cumulative Trauma Disorders” OR “Ligaments, Articular/injuries” OR “Lacerations”)) AND ((“Shoulder” OR “Shoulder Injuries” OR “Shoulder Pain/physiopathology” OR “Shoulder Pain/epidemiology” OR “Shoulder Pain/etiology” OR “Rotator Cuff Injuries” OR “Shoulder Dislocation” OR “Shoulder Fractures” OR “Bankart Lesions” OR “Shoulder Impingement Syndrome”)) AND ((“Water Sports” OR “Wave Surfing” OR “Surfing, Wave” OR “Surfboarding” OR “Surfing” OR “Surf”)) (Word variations have been searched)”	**4**
